# Extending the Three-Dimensional Culture of Adipocytes Through Surface Coatings

**DOI:** 10.3390/bioengineering12030266

**Published:** 2025-03-08

**Authors:** Sheetal Chowdhury, Komal Beeton, Zacchaeus Wallace, Maggie Moore, Gene L. Bidwell, Amol V. Janorkar

**Affiliations:** 1 Department of Biomedical Materials Science, University of Mississippi Medical Center, 2500 N State Street, Jackson, MS 39216, USA; 2 Department of Cell and Molecular Biology, University of Mississippi Medical Center, 2500 N State Street, Jackson, MS 39216, USA; 3 Department of Neurology, University of Mississippi Medical Center, 2500 N State Street, Jackson, MS 39216, USA

**Keywords:** three-dimensional spheroids, elastin-like polypeptide, RGD, spheroid retention

## Abstract

To mimic the important features of progressing adiposity, in vitro adipose cell culture models must allow gradual intracellular fat accumulation in the three-dimensional (3D) arrangement of adipose-derived stem cells (ASCs) over a long-term culture period. Previously, elastin-like polypeptide (ELP) and polyethyleneimine (PEI) have been used to culture human adipose-derived stem cells (hASCs) as 3D spheroids and to differentiate them to adipocytes over a relatively long culture period of up to 5 weeks. In this study, to further enhance the spheroid adhesion properties, ELP was fused with Arginine–Glycine–Aspartic Acid (RGD) residues, known for their role as cell-attachment sites. This study aimed to assess whether the addition of RGD to the C-or N-terminus of ELP would impact the spheroid-forming ability of ELP-PEI coatings. ELP-RGD conjugates were produced using genetically modified *Escherichia coli* to express ELP-(RGD)_3_ and (RGD)_3_-ELP, followed by chemical conjugation with PEI. SDS gel electrophoresis, FTIR spectroscopy, and turbidimetry analyses revealed that ELP was conjugated with RGD without much alteration in the molecular weight, functional groups present, and transition temperature of ELP. The addition of RGD to ELP also did not affect the chemical conjugation capacity of ELP to PEI. We observed that the ELP-PEI coating formed slightly larger spheroids (61.8 ± 3.2 µm) compared to the ELP-(RGD)_3_-PEI and (RGD)_3_-ELP-PEI coatings (56.6 ± 3.0 and 53.4 ± 2.4 µm, respectively). Despite the size difference, ELP-(RGD)_3_-PEI coatings exhibited superior spheroid retention during media changes, with minimal spheroid loss. DNA assay results confirmed a significant decrease in the DNA concentration (*p* < 0.05) after the 20 media changes for spheroids cultured on the ELP-PEI coating, indicating spheroid loss. However, there was no significant difference in DNA concentration before and after 20 media changes for spheroids cultured on the ELP-(RGD)_3_-PEI and (RGD)_3_-ELP-PEI coatings (*p* > 0.05). These findings suggest that RGD incorporation does not hinder the initial spheroid formation ability of the ELP-PEI coating and enhances spheroid retention under dynamic culture conditions.

## 1. Introduction

Almost 40% of the adult population in the United States has obesity. Obesity serves as a catalyst for a cascade of health issues, including diabetes, cardiovascular ailments, and cancer. Recent studies have highlighted the importance of targeting adipose tissue metabolism as a strategy to combat obesity, with emerging research focusing on the role of white adipose tissue (WAT) browning as a potential therapeutic approach [1]. Nearly all FDA-approved anti-obesity drugs act as appetite suppressors and satiety inducers [2]. To identify better drug candidates that restore the healthy metabolic function of the adipose tissue, and to define the underlying mechanisms of action of such drug candidates on the adipose tissue metabolism and function, researchers have developed several in vitro cell culture models. In vitro models seek to investigate the responses of adipocytes (the primary cell type in fat tissue) under fat-laden states. The understanding gained from in vivo-like culture models will allow us to develop more effective therapeutics to treat obesity and its disastrous cardiovascular sequelae. However, when exposed to pathophysiological conditions, optimal adipose-specific responses can be expected from an in vitro adipocyte model only if it closely resembles the in vivo tissue structure.

Various in vitro culture techniques are available to differentiate human adipose-derived stem cells (hASCs) into mature adipocytes. First is the culture of hASCs on a tissue culture plastic surface, forming a 2D monolayer. However, 2D culture fails to mimic the in vivo morphology because the adipocytes in the in vivo adipose tissue are present in a clustered form. Another emerging technique is to culture the hASCs in the form of 3D spheroids, which mimic the adipose tissue through having clustered cells and less extracellular matrix. Spheroids, the three-dimensional clusters of cells, thus closely resemble the in vivo tissue structure and can potentially recapitulate the pathophysiological conditions. These models allow for better cell–cell and cell–ECM interactions, making them more physiologically relevant for studying lipid metabolism and drug responses [3]. There are various ways to culture the 3D spheroids: hanging drop, non-adherent surface, spinner culture, etc. Each of the aforementioned methods has its own drawbacks. In the case of the hanging drop technique, only a single spheroid can be cultured per droplet, spheroids need to be transferred into another well-plate for post-processing (for example, performing any assay), and any evaporation of the small volume of media used (5 to 20 µL per droplet) can prevent long-term culture [4]. For non-adherent surfaces, there is the probability of the removal of the spheroids while changing the media. The cells are exposed to continuous shear stress for spinner cultures, making them functionally impaired. As a result, adipocytes in the existing 3D models fail to achieve a physiologically relevant outcome of high triglyceride accumulation (i.e., maximal adiposity) over a long-term culture period [5,6].

Our previous studies used elastin-like polypeptide (ELP) and polyethyleneimine (PEI) copolymers to form in vitro 3D spheroid adipocyte cultures [5,7,8,9]. ELP has inducible temperature-dependent phase transition behavior, which can be modified with chemical moieties. We have shown that the ELP-PEI coating forms a stable anchor promoting the self-aggregation of adipocytes into spheroids and that the spheroids achieve significant triglyceride accumulation [5]. In our previous studies, we have shown that 3D spheroid cultures exhibit higher lipid accumulation, altered gene expression, and enhanced adipogenic differentiation compared to traditional 2D monolayers. These findings highlight the advantages of the 3D spheroid model, which more closely resembles native adipose tissue morphology and function, making it a superior platform for studying adipose metabolism and drug responses [7,8,10]. However, gradual spheroid loss occurs over a long-term cell culture period due to the necessary repeated media changes, which limits the applicability of this culturing technique. Therefore, a coating that can achieve minimum spheroid loss to support a long-term culture of adipocytes is urgently needed. The tri-amino acid sequence, arginine–glycine–aspartic acid, or “RGD,” is the most extensively researched adhesive peptide in the biomaterials sector [11]. RGD is the key integrin-binding domain present within extracellular matrix (ECM) proteins such as fibronectin, vitronectin, fibrinogen, osteopontin, and bone sialoprotein. The RGD sequence can bind to cell surface integrins and encourage the attachment of various cell types [12]. In this work, we incorporated the cell adhesive peptide RGD at the C- or N-terminus of ELP via recombinant engineering from *Escherichia coli* (*E. coli*) as a feasible and inexpensive approach to improve spheroid adhesion and retention. We hypothesized that introducing the RGD cell adhesive domains in the ELP structure would achieve more robust spheroid surface attachment.

## 2. Materials and Methods

**Figure 1:**
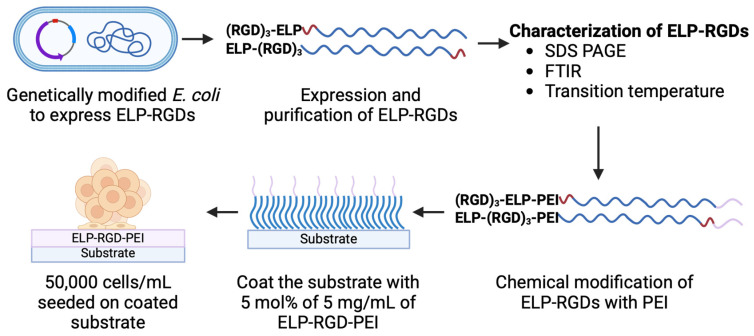
Schematic representation of the process flow for engineering ELP-RGD-PEI functionalized substrates.

Figure 1 shows a schematic representation of the process flow for engineering ELP-RGD-PEI functionalized substrates, illustrating the key steps involved in their fabrication and functionalization.

### 2.1. Expression and Purification of ELP and ELP-RGDs

An ELP containing 40 pentapeptide repeats with valine in the guest residue position (VPGVG)_40_ was used. Three sequences of RGD were attached to the N- or C-terminus of ELP, forming the (RGD)_3_-ELP and ELP-(RGD)_3_. The orientation of the RGD sequence was chosen to investigate how terminal positioning influences spheroid formation and retention. Coding sequences for (RGD)_3_-ELP and ELP-(RGD)_3_ were generated by DNA synthesis using codons optimized for expression in *E. coli* (Invitrogen GeneArt). The peptide sequences of the ELP constructs are listed in Table 1. The coding sequences were excised from the pMA cloning vector and ligated into the pET-25b+ expression vector between the NdeI and BamHI restriction sites, and the constructs were confirmed by DNA sequencing. The pET25+ vectors containing the ELP, (RGD)_3_-ELP, or ELP-(RGD)_3_ coding sequences were transformed into *E. coli* BLR(DE3) (Novagen (EMD), Madison, WI) and selected on ampicillin agar plates. The liquid cultures (Terrific Broth, MP Biomedicals) were inoculated and grown overnight, then further cultured in a shaker flask at 37 °C for 24 h. The bacteria were harvested and lysed by sonic disruption, and the cell debris was removed using centrifugation at 4 °C. Nucleic acids were precipitated with polyethyleneimine (PEI) treatment followed by centrifugation at 4 °C. Next, the ELPs were purified using the inverse phase transition behavior of ELP. The ELP was precipitated out of the solution above its phase transition temperature by raising the salt concentration to 1M NaCl and heating the solution at 40 °C, followed by centrifugation. The purification of ELPs was carried out in repeated cycles of dissolving ELP in PBS at 4 °C followed by precipitation. This cycle was repeated three times until the purified protein was obtained. The purity of the proteins was assessed by SDS-PAGE (Sodium Dodecyl Sulfate–Polyacrylamide Gel Electrophoresis). The ELP solution was dialyzed against deionized water using a regenerated cellulose membrane (molecular weight cut-off = 3500 Da), and the purified ELPs were then lyophilized.

### 2.2. Characterization of ELP and ELP-RGD

ELP, ELP-(RGD)_3_, and (RGD)_3_-ELP were analyzed by attenuated total reflectance Fourier transform infrared spectroscopy (ATR-FTIR) using a Spectrum 100 FTIR spectrometer (Perkin-Elmer, Waltham, MA, USA), and scanned from 650 to 4000 cm^−1^ using a diamond/ZnSe crystal at a resolution of 4 cm^−1^ to detect the functional groups present. ELP, ELP-(RGD)_3_, and (RGD)_3_-ELP were assessed for their molecular weights by binding them with sodium dodecyl sulfate and running them on a polyacrylamide gel (SDS-PAGE). The gel was negatively stained with a copper stain to visualize the resulting bands. The temperature-induced aggregation above the inverse phase transition temperature (T_t_) for the ELP, ELP-(RGD)_3_, and (RGD)_3_-ELP was characterized by monitoring absorbance at 350 nm as a function of temperature. Briefly, 5 mg/mL of the ELP, ELP-(RGD)_3_, and (RGD)_3_-ELP solutions in phosphate buffer (1 mmol/L Na_2_PO_4_, 0.2 mmol/L KH_2_PO_4_, 0.27 mmol/L KCl, 13.7 mmol/L NaCl) were heated or cooled at a constant rate of 1 °C/min in a temperature-controlled multicell holder in a UV-visible spectrophotometer (Cary 100, Varian instruments) from 20 °C to 60 °C. The derivative of the absorbance at A_350_ was plotted against the temperature. The temperature where the slope was at its maximum was defined as the T_t_.

### 2.3. Chemical Modification of ELP and ELP-RGDs with PEI

Using carbodiimide chemistry, the ELPs were modified with PEI (Mol. wt. = 800) via an EDC-NHS coupling reaction. A total of 30 mg of ELPs were mixed in MES buffer (0.1 M MES, 0.5 M NaCl, pH 6.2) with a 10-molar excess of PEI, EDC, and NHS. The pH of the mixture was adjusted to 6.8 using 1 N HCl and NaOH, and the contents were allowed to react with each other overnight at 4 °C. The unreacted PEI was then removed using the inverse phase transition purification method. Note that since the RGD repeat sequences were added to the C-terminus of ELP in ELP-(RGD)_3_, the -COOH of RGD took part in the reaction with PEI. Similarly, for (RGD)_3_-ELP, the RGD repeat sequences were added to the N-terminus of ELP; therefore, the -COOH end group ELP reacted with PEI.

### 2.4. Characterization of Chemically Conjugated ELP-PEI and ELP-RGD-PEIs

The ELP concentration in the ELP-PEI and ELP-RGD-PEIs solutions were measured using a Nanodrop UV-Vis spectrophotometer (ND-1000, Thermo Scientific, Waltham, MA, USA). Then, the o-phthalaldehyde (OPA) assay was used to measure the percent conjugation of ELP and PEI following the EDC-NHS coupling reaction. Equivalent molar concentrations of unreacted ELP, PEI, and conjugated ELP-PEI, ELP-RGD_3_-PEI, and RGD_3_-ELP-PEI were mixed with the OPA assay and measured using a fluorescence plate reader (Biotek FL×800, Winooski, VT, USA) with the excitation and emission filters set at 360 and 460 nm, respectively. The emission intensity of the conjugates was compared to the emission intensity of ELP, representing 0 mol% conjugation, and that of PEI, representing 100 mol% conjugation.

### 2.5. Modification of Tissue Culture Polystyrene (TCPS)

ELP-PEI, ELP-(RGD)_3_-PEI, and (RGD)_3_-ELP-PEI were adsorbed on 24-well TCPS plates using 200 µL of 5 mg/mL, with 5 mol% of conjugate solution in each well. The plates were placed inside the incubator for 48 h at 37 °C to remove the solvent.

### 2.6. hASCs Isolation and Induction of Spheroid Formation

We utilized discarded anterolateral thigh (ALT) flap adipose tissue from an adult female patient following the protocol approved by the University of Mississippi Medical Center Institutional Review Board (Approval # 2012–0004). The adipose tissue was dissected and digested to isolate undifferentiated ADSCs for further culturing, following the techniques demonstrated by Flynn and adapted from the methods established by Hauner et al. [13,14,15]. Briefly, the adipose tissue was collected in 1× DMEM, which was immediately delivered to the laboratory. Working under a sterile laminar-flow hood, the tissue was placed in a tissue culture dish, minced into small (∼1 mm^3^) sections, and washed with PBS to help remove blood, oil, serum, other vascular components, and lymph vessels. The tissue fragments were digested in Liberase TM collagenase (0.12 AU/mL) in PBS supplemented with 3 mm glucose, 25 mm HEPES, and 20 mg/mL BSA for 30 min at 37 °C in a shaker bath. The tissue was then filtered through a 100 μm nylon mesh into a fresh 50 mL tube to remove undigested fragments. Whole media (50:50 DMEM: Ham’s F12, 10% calf serum, pen/strep) was added to the filtrate, and the cells were allowed to separate by gravity. The supernatant was aspirated to remove mature adipocytes, and the remaining filtrate was centrifuged at 1200× *g* for 5 min. The cell pellet was suspended in erythrocyte lysis buffer (155 mM NH_4_Cl, 10 mM KHCO_3_, and 0.1 mM EDTA) at room temperature for 10 min. The remaining cells were pelleted again, resuspended in whole media (DMEM + 10% calf serum), and filtered through a 70 μm mesh nylon net to remove mature adipocytes, clusters of erythrocytes and endothelial cells, and any remaining tissue and cell aggregates. The remaining stromal vascular fraction, including hASCs, was cultured on TCPS dishes in 50:50 DMEM: Ham’s F12 with 10% calf serum at 37 °C and 5% CO_2_, with regular media changes every 2-3 days. Culturing the cells on TCPS dishes facilitated the selective attachment and proliferation of hASCs, as confirmed by the identification of mesenchymal stem cell surface markers [16]. A total of 50,000 hASCs were seeded in each well of a 24-well plate and allowed to form spheroids over a 72 h culture period.

### 2.7. Measurement of Spheroid Size

Spheroid size was assessed using an Olympus IX 81 optical microscope (Olympus, Center Valley, PA, USA) with a total magnification of 100×. For the image analysis using ImageJ software (Version 1.54h), spheroids (*n* > 50) were analyzed for each type of coating. The average spheroid size and spheroid size distribution were measured for each coating type.

### 2.8. Frequent Media Change Assay

To determine the coating with an improved spheroid retention capability and to recapitulate the long-term culture, we used an in-house-developed frequent media change assay. In this assay, we captured the image of the entire well after forming the spheroids. The media was then replaced 20 times, and the image of the entire well was captured again. The image analysis was performed to obtain a qualitative insight into any spheroid movement and/or loss after the 20 media changes. To obtain a quantitative result for the spheroid loss, a DNA assay was performed using CyQUANT Cell Proliferation Assay Kit and Fluorescence Assay using the manufacturer’s protocol. The DNA concentration of the spheroids before and after the 20 media changes was measured.

### 2.9. Statistical Analysis

Statistical analysis of the results was performed through one-way ANOVA with a Games–Howell *post hoc* test designed for unequal variances, and a Bonferroni *post hoc* test for equal variances. The data are presented as the mean ± 95% confidence interval, with significance attributed to values where *p* ≤ 0.05.

## 3. Results

**Figure 2:**
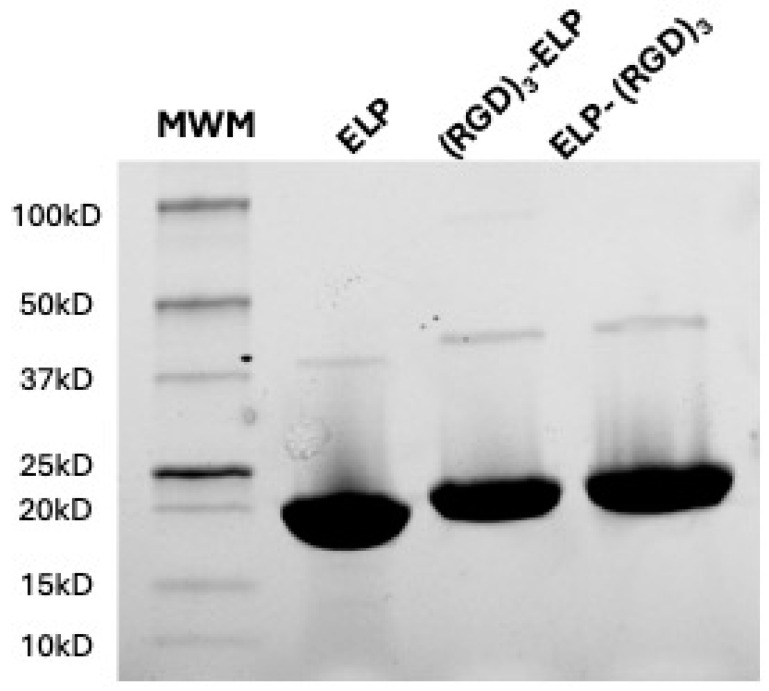
SDS-PAGE analysis of the synthesized polypeptides ELP, ELP-(RGD)_3_, and (RGD)_3_-ELP at a 50 µg polymer concentration to assess the molecular weights. A molecular weight marker (MWM) was included in the gel to estimate the molecular weights of the purified proteins. The black dot is an artifact of the imaging process.

The ELP-(RGD)_3_ and (RGD)_3_-ELP were successfully expressed in *E. coli*, isolated, and purified utilizing the inverse phase transition behavior of ELP. The synthesized polypeptides were run in the SDS-PAGE to analyze the molecular weights of the proteins. ELP, ELP-(RGD)_3_, and (RGD)_3_-ELP were observed to produce a single band for 50 µg of the polymer with a molecular weight in the range of 15 to 20 kDa, as shown in Figure 2. A visible dimer band is present in the range of 37 to 50 kDa, corresponding to the disulfide-linked dimers of two ELP molecules. These dimers result from incomplete reduction prior to gel electrophoresis, a common occurrence with constructs containing a single cysteine residue.

**Figure 3:**
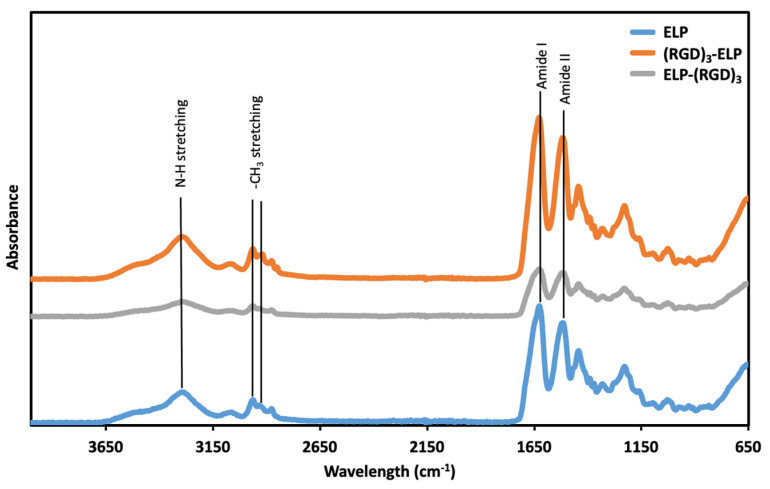
ATR-FTIR spectra analysis of ELP and ELP-RGDs, providing insights into the molecular composition and structural characteristics of the engineered elastin-like polypeptides functionalized with RGD peptides.

The functional groups of ELP, ELP-(RGD)_3_, and (RGD)_3_-ELP were assessed using ATR-FTIR. In the ATR-FTIR spectrum (Figure 3), peaks at wavenumbers 3200 and 3000 cm^−1^, corresponding to the N–H stretching vibrations, were observed for ELP, ELP-(RGD)_3_, and (RGD)_3_-ELP. A peak at 1632 cm^−1^, corresponding to the C = O stretches in the amide functionality (amide I peak), and a peak at 1512 cm^−1^, which is the combination band of the N–H bending and C–N stretching vibrations (amide II peak), were also observed for ELP, ELP-(RGD)_3_, and (RGD)_3_-ELP. Similarly, ELP, ELP-(RGD)_3_, and (RGD)_3_-ELP showed peaks at around 2969 cm^−1^ and 2922 cm^−1^, corresponding to -NH_2_ stretching. Therefore, the ATR-FTIR spectroscopy showed that the ELP-(RGD)_3_ and (RGD)_3_-ELP had chemical signatures equivalent to the ELP.

**Figure 4:**
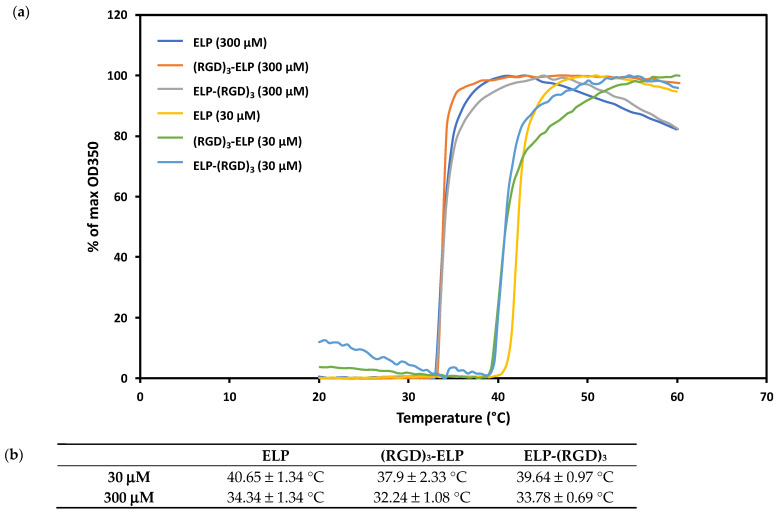
(**a**) Concentration-dependent transition temperature assessed by turbidity analysis for ELP, ELP-(RGD)_3_, and (RGD)_3_-ELP, revealing the impact of concentration on the phase transition behavior of the engineered biomaterials. (**b**) Table displaying the phase transition temperatures of ELP, ELP-(RGD)_3_, and (RGD)_3_-ELP. The addition of the RGD peptide did not result in a significant change in transition temperature (*p* > 0.05), whereas increasing concentration led to significant differences (*p* < 0.05).

We observed that the transition temperature for ELP at a concentration of 30 μM was 40.7 ± 1.3 °C, whereas ELP at a concentration of 300 μM exhibited a transition temperature of 34.3 ± 1.3 °C (Figure 4). The transition temperatures for (RGD)_3_-ELP and ELP-(RGD)_3_ at 30 μM were observed to be 37.9 ± 2.3 °C and 39.6 ± 1.0 °C, respectively, whereas the transition temperatures for (RGD)_3_-ELP and ELP-(RGD)_3_ at 300 μM were observed to be 32.2 ± 1.1 °C and 33.8 ± 0.7 °C, respectively.

The conjugation efficacy was quantified using an OPA assay that was used to measure the primary amine content of ELP before and after conjugation with PEI. To calculate the percent of conjugation, several molar concentrations of pure ELP, unreacted PEI, and ELP–PEI conjugates were assayed and read with a fluorescence plate reader. The resulting fluorescent emissions were plotted against molar concentration, and the line of best fit was plotted for each. The slope of the best-fit line for the corresponding pure ELP was used as a baseline, and the ratio of the slope of the best-fit line for the related ELP–PEI, (RGD)_3_-ELP-PEI, and ELP-(RGD)_3_-PEI conjugate to that for the unreacted PEI was used to determine the proportion of PEI-bound ELP molecules after the reaction. It was observed that the ELP-RGDs were successfully conjugated with PEI with similar conjugation percentages to that of the neat ELP equivalent to 11–12 mol % (Table 2); thus, the addition of RGD did not affect the conjugation capability of ELP with PEI (*p* > 0.05).

**Figure 5:**
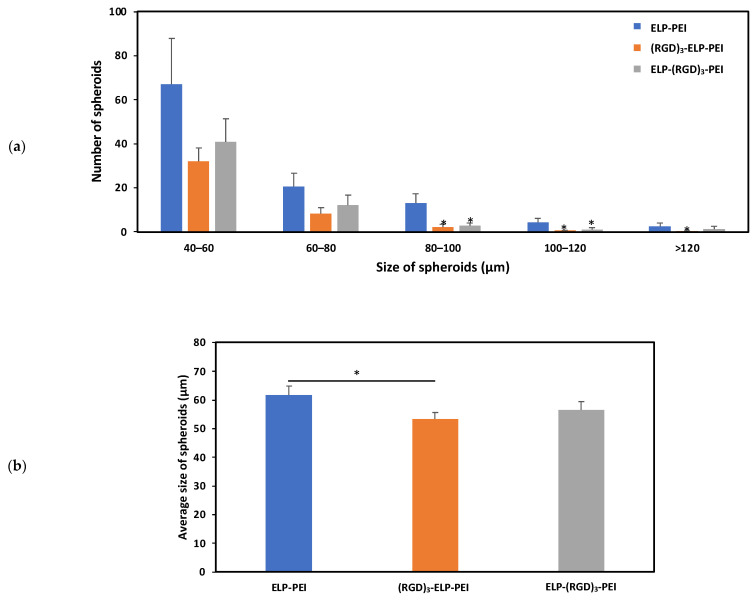
Influence of adding RGD to ELP on spheroid formation, depicting (**a**) the size distribution of spheroids formed on different coatings. * represents *p* ≤ 0.05 compared to the ELP-PEI coating for the respective size group. (**b**) Average size of the spheroids formed on different coatings. * represents *p* ≤ 0.05 compared to the ELP-PEI coating.

To investigate the potential of the (RGD)_3_-ELP-PEI and ELP-(RGD)_3_-PEI conjugates to form spheroids, hASCs were cultured on a control ELP-PEI coating and the experimental (RGD)_3_-ELP-PEI and ELP-(RGD)_3_-PEI coatings. After a period of 72 h, it was observed that the hASCs organized themselves into spheroids on all the coatings. Figure 5b illustrates the difference in the spheroid diameter observed for the ELP-PEI, (RGD)_3_-ELP-PEI, and ELP-(RGD)_3_-PEI coatings. The average sizes of the spheroids for the ELP-PEI, (RGD)_3_-ELP-PEI, and ELP-(RGD)_3_-PEI substrates were observed to be 61.8 ± 3.2 µm, 53.4 ± 2.4 µm, and 56.6 ± 3.0 µm, respectively. The measured spheroid diameter exhibited equal variance, warranting the use of the Bonferroni *post hoc* test. The average spheroid diameter for (RGD)_3_-ELP-PEI was significantly different (*p* < 0.05) compared to ELP-PEI.

**Figure 6:**
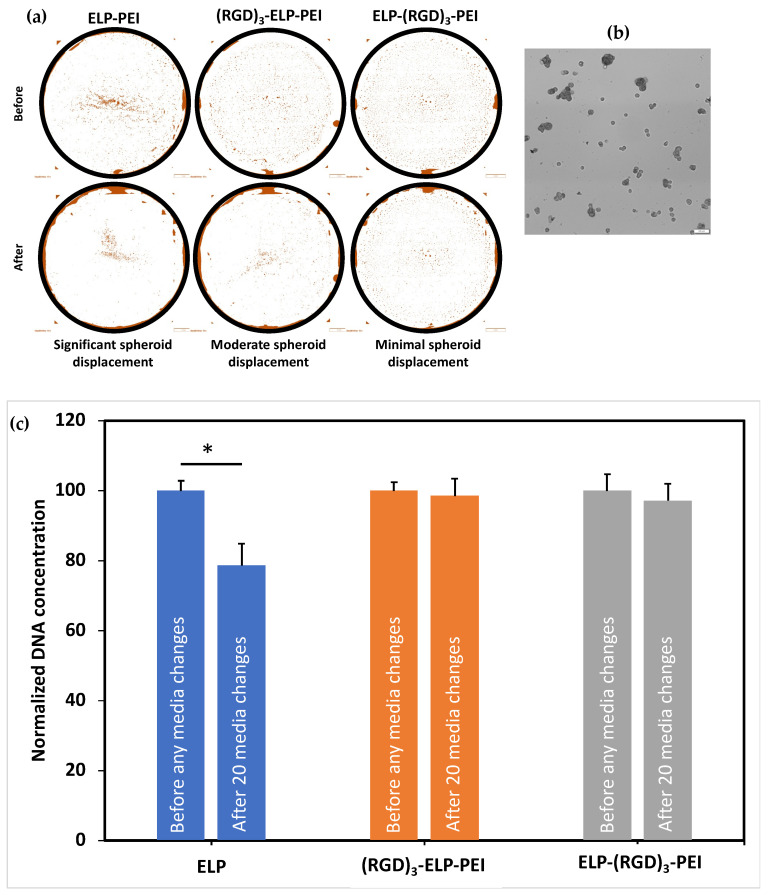
Assessment of the effect of adding RGD to ELP on spheroid stability through a frequent media change assay. (**a**) Representative optical microscopy images of culture wells before and after 20 media changes. *n* = 3 coatings. (**b**) Representative image of spheroids cultured on the ELP-RGD-PEI-coated plates. (**c**) DNA content of the cultures before and after 20 media changes. * represents *p* ≤ 0.05 compared to the ELP-PEI coating.

To visualize the effect of RGD on spheroid retention due to its cell adhesive properties, the image of the entire well of the 24-well plate was captured using an Olympus optical microscope before and after 20 media changes. Differences in the distribution of spheroids on the various coatings before and after the media changes revealed that the ELP-PEI coating showed significant spheroid displacement, the (RGD)_3_-ELP-PEI coating showed moderate spheroid displacement, and the ELP-(RGD)_3_-PEI coating supported better spheroid retention with minimal spheroid displacement (Figure 6a). To quantify the spheroid loss, we measured the DNA concentration of the cells before and after the media changes. The DNA concentrations measured for the ELP-PEI coating before and after 20 media changes were 15.55 ± 0.41 ng/mL and 12.23 ± 0.90 ng/mL, respectively. The DNA assay results, shown in Figure 6b, revealed that the ELP-PEI coating suffered a significant spheroid loss, as indicated by the decrease in the DNA content after the 20 media changes (*p* < 0.05). The DNA concentrations measured for the (RGD)_3_-ELP-PEI coating before and after 20 media changes were 14.87 ± 0.34 ng/mL and 14.64 ± 0.70 ng/mL, respectively. The DNA concentrations measured for the ELP-(RGD)_3_-PEI coating before and after 20 media changes were 13.18 ± 0.58 ng/mL and 12.81 ± 0.59 ng/mL, respectively. There was no significant difference in DNA content before and after 20 media changes observed for the ELP-(RGD)_3_-PEI and (RGD)_3_-ELP-PEI coatings (*p* > 0.05). The optical microscopy and DNA analysis data for the frequent media change assay revealed that the ELP-(RGD)_3_-PEI coating supported better spheroid retention with minimal spheroid movement.

## 4. Discussion

ELPs are a family of polypeptides with a repeating structure derived from mammalian elastin, valine–proline–glycine–X–glycine, where X = guest amino acid [17]. Being biocompatible, resorbable, and non-immunogenic, ELPs have been used for drug delivery and tissue engineering [18,19,20,21,22,23]. The recombinant production of ELPs by *E. coli* allows complete control over their structure and molecular weight with a relatively simple purification method due to their inverse phase transition behavior [24]. Janorkar et al. have successfully used ELP conjugated to amine-containing polyelectrolytes to form 3D spheroids. Here, the polyelectrolyte induces spheroid formation, while the ELP moiety helps to tether the spheroids to a substrate. Janorkar et al. created an array of aminated-ELP copolymers by varying the polyelectrolyte structure and they extended the 3D adipocyte culture period to 5 weeks [7,8,9,10]. In the current study, repeat sequences of arginine–glycine–aspartic acid (RGD) were added to the N- and C-terminus of ELP, forming (RGD)_3_-(VPGVG)_40_ and (VPGVG)_40_-(RGD)_3_ via recombinant engineering from *E. coli*. We hypothesized that introducing the RGD cell adhesive domains in the ELP structure will not affect the phase transition behavior of ELP, but will reduce the amount of spheroid loss due to the interactions between the cell surface integrins and the RGD motifs.

Compared to the complete ECM proteins, synthetic RGD peptides have several benefits for use as biomaterials. In general, complete ECM proteins need to be isolated from organisms and purified. Another advantage of the RGD peptides is that the functionality of RGD is typically maintained throughout the processing and sterilization steps necessary for the synthesis of biomaterials. When compared to using xenograft or cadaveric protein sources used for implant application, the use of RGD peptides reduces the risk of immune reactivity or pathogen transfer. RGD peptides are more cost-effective and easier to synthesize, which makes clinical translation easier. Finally, controlled densities and orientations of RGD peptides can be coupled to material surfaces [11,12,25].

The neat (VPGVG)_40_ ELP was compared with the (RGD)_3_-containing ELPs for molecular weight using SDS-PAGE, chemical signature using FTIR spectroscopy, phase transition behavior using turbidimetry, and conjugation capability with PEI using OPA analysis. SDS-PAGE was used to qualitatively assess if there was a significant change in the molecular weight of the ELP construct after conjugation with (RGD)_3_. The observed molecular weights of ELP-RGDs were similar to the observed molecular weight of ELP, as shown in Figure 2. This indicated that the addition of RGDs to the ELP did not significantly change the molecular weight of the polypeptide. The theoretical molecular weight of the ELP construct (VPGVG)_40_ is 17 kDa, whereas the molecular weight of RGD is 346 Da. Therefore, the addition of (RGD)_3_ to (VPGVG)_40_ would increase the molecular weight of the constructs by only 1 kDa. This increase in the molecular weight is not significant enough to be seen in the SDS-PAGE gel we used. In ATR-FTIR, it was observed that ELP, ELP-(RGD)_3_, and (RGD)_3_-ELP had similar chemical signatures (Figure 3). This similarity is likely attributable to RGD and ELP both being composed of amino acids and having similar chemical functional groups present. Additionally, the relatively low concentration of the RGD peptide within the overall ELP sequence likely results in minimal spectral differences, as the dominant signals arise from the ELP backbone.

Thermal responsiveness is a distinguishing feature of ELPs. ELPs exhibit low critical solution temperature (LCST) phase behavior, which is an entropically driven phenomenon that leads polypeptide solutions to become insoluble above their phase transition temperature (T_t_) [26,27]. The Gibbs free energy change is negative at low temperatures, allowing ELPs and solvents to mix spontaneously. Mixing becomes more energetically unfavorable as the temperature rises, causing the ELP to phase separate from water [28,29]. The temperature at which this phase separation occurs is determined by several factors, one of them being the concentration of the polypeptide solution. At temperatures below the T_t_, the hydrophobic ELP domains are shielded by clathrate water, which prevents the self-aggregation of the polypeptide. At higher temperatures, the hydrogen bonds between the clathrate water molecules are disrupted, which then exposes the hydrophobic domains for self-interaction. At higher concentrations of ELP, the number of water molecules forming the clathrate is relatively lower. Therefore, the hydrogen bonds can be disrupted at a lower temperature, leading to a reduced T_t_ [30]. In line with these principles, we observed that as the solution concentration increased, the transition temperature of ELP decreased, as shown in Figure 4. Molecular weight is an intrinsic parameter that controls the transition behavior of ELP, where the transition temperature is inversely related to large increases in molecular weight. The increase in the molecular weight of ELP constructs by 1 kDa upon the addition of RGD did not affect the inverse phase transition behavior of ELP, similar to the results reported by MacEwan et al. [31]. The thermal behavior of ELP is also governed by any hydrophilic amino acid present in the guest residue position of VPGXG, and in general, the presence of hydrophilic residues decreases the temperature responsiveness of ELP, i.e., increases the T_t_. While RGD is hydrophilic in nature, we did not observe any significant effect of the addition of RGD residues on the phase transition behavior of ELP. The coating process was performed at 37 °C to enhance aggregation and adhesion to the substrate, ensuring better stability. More importantly, because the T_t_ values were lower than the cell culture temperature (37 °C), the ELP-PEI, (RGD)_3_-ELP-PEI, and ELP-(RGD)_3_-PEI stayed securely coated atop the TCPS surface and did not dissolve back into the culture medium during the cell culture time. In a previous study, a 300 μM (5 mg/mL) concentration of ELP-PEI demonstrated superior spheroid formation and retention capability, suggesting that this concentration is optimal for maintaining coating stability while supporting cell attachment and biological function [7].

PEI is a branched polyelectrolyte with a primary/secondary/tertiary amine ratio of 1:4:1 and approximately five primary amine groups per molecule. We chemically conjugated PEI with ELP and ELP-RGD via a carbodiimide chemical coupling reaction. RGD addition to ELP did not affect the PEI conjugation process using carbodiimide chemistry (Table 2). This may be attributed to there being no significant changes in molecular weight or functional group composition, indicating that the structure and essential chemical reactions were preserved. This suggests that incorporating RGD into ELP constructs is feasible without compromising the efficiency of PEI conjugation.

The hASCs were seeded on the coated plates and allowed to form spheroids for 3 days and then imaged using an optical microscope, followed by image analysis using ImageJ. It was observed that the spheroid-inducing capability of the ELP-PEI was not altered by the addition of (RGD)_3_ to ELP, as shown in Figure 5. Spheroid size is influenced by multiple factors, including initial cell seeding density, culture duration, and the properties of the substrate or coating material. To ensure that the variations in spheroid size observed in this study were primarily due to the effects of the different coatings, we controlled for the initial cell density by seeding all cultures at equal concentrations and maintaining them under identical culture conditions for the same duration. By standardizing these parameters, we minimized potential confounding effects, allowing us to attribute differences in spheroid formation and retention to the biochemical and biophysical properties of the ELP-based coatings. This approach ensured a more accurate assessment of how coating modifications impact spheroid aggregation, attachment, and long-term stability. The size of the spheroids formed using the substrates compared well with the other approaches to generating spheroids. It should be noted that an average spheroid diameter close to 100 µm is suitable for cell viability and functionality as it does not hinder oxygen and nutrient transport. Previous studies have demonstrated that adipocyte function, including lipid accumulation, metabolic activity, and differentiation, remains largely unaffected by small changes in spheroid size [8,32,33,34,35,36].

**Figure 7:**
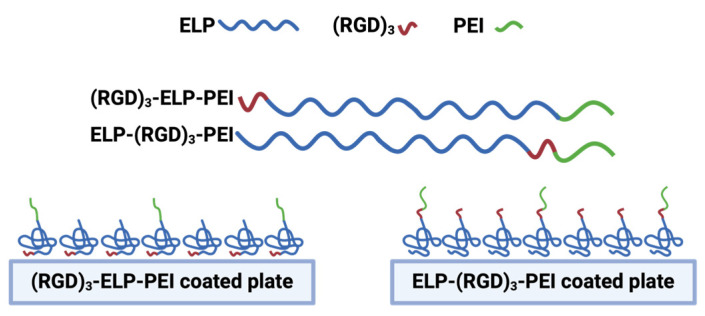
Schematic representation of ELP-(RGD)_3_-PEI and (RGD)_3_-ELP-PEI adsorbed on the surface. (RGD)_3_ at the N-terminus is away from PEI and farther away from the aqueous environment, limiting cell exposure. (RGD)_3_ at the C-terminus is near PEI and exposed to the aqueous environment, improving spheroid interaction. Thus, ELP-(RGD)_3_-PEI demonstrates superior peptide presentation for enhanced spheroid adhesion with minimal displacement.

Cell viability is a critical factor in ensuring that observed changes in DNA concentration accurately reflect spheroid retention rather than cell death or apoptosis. To assess the viability of the adipocyte spheroids in our system, live/dead staining has been performed in previous studies [9,10]. These studies demonstrated that 3D adipocyte spheroids cultured on ELP-PEI coatings maintained a high cell viability, with minimal cell death observed under our experimental conditions. Furthermore, in Turner et al. (2015) [10], a live/dead assay was used to compare 2D monolayer cultures and 3D spheroids, confirming that the 3D spheroid model remained viable over extended culture periods. Based on these previous findings, we used DNA quantification as a reliable measure of spheroid retention in the current study. Given the established viability of the spheroids in our system, the DNA concentration data accurately reflected spheroid stability rather than potential cell loss due to apoptosis. The incorporation of RGD into a biomaterial has been proven to be an effective tool to enhance cell attachment and tissue regeneration. RGD specifically interacts with integrin receptors with a high affinity for certain integrin subtypes such as αvβ_3_ and α_5_β_1_, thereby allowing for targeted cell adhesion and enabling biomaterials to selectively interact with specific cell types. From the frequent media change assay, it was observed that the addition of RGD into the coating, i.e., ELP-(RGD)_3_-PEI and (RGD)_3_-ELP-PEI, resulted in better spheroid retention compared to the neat ELP-PEI (Figure 6c). Moreover, ELP-(RGD)_3_-PEI showed minimal spheroid displacement in comparison to (RGD)_3_-ELP-PEI (Figure 6a), suggesting the potential influence of the RGD positioning at the N- or C-terminus of aminated-ELP. We explain this interesting result based on the knowledge that proteins undergo a series of conformational changes during protein adsorption. Consequently, the adsorption of ELP-(RGD)_3_-PEI and (RGD)_3_-ELP-PEI on the surface may lead to a series of conformational changes resulting in variations in the exposed peptide sequences capable of interacting with cells. The ELP exhibits hydrophobic properties, while PEI possesses hydrophilic properties. Consequently, when introduced to a substrate for coating formation, the hydrophobic nature of ELP induces its folding through hydrophobic interactions, while PEI attached to the C-terminus of ELP is prominently exposed to the surrounding aqueous environment. In (RGD)_3_-ELP-PEI, the (RGD)_3_ is attached to the N-terminus of ELP and the PEI is attached to the C-terminus of ELP. The (RGD)_3_ will thus be farther away from the aqueous environment, which would hinder its exposure to cells for interaction, as shown in Figure 7. The (RGD)_3_ is attached to the C-terminus and next to PEI in ELP-(RGD)_3_-PEI. This molecular arrangement would allow the (RGD)_3_ to remain exposed to the aqueous environment, resulting in the improved adhesion of the spheroids.

The incorporation of the RGD peptide into biomaterials not only enhances cell attachment but may also activate specific signaling pathways that influence changes in cell behavior. These pathways, mediated by the interaction of RGD with integrin receptors, could potentially lead to alterations in cell morphology, proliferation, or differentiation over time. To validate these effects, long-term culture studies are necessary, as they would allow for the observation of sustained cellular responses and provide further insights into the impact of RGD on cellular functions, beyond initial adhesion.

While this study demonstrates the effectiveness of ELP-(RGD)_3_-PEI coatings in enhancing adipocyte spheroid retention, several limitations must be considered. One key limitation is the inherent complexity of translating in vitro spheroid models to in vivo systems. Although the 3D spheroid culture more closely mimics the architecture and cellular interactions of native adipose tissue compared to traditional 2D cultures, it does not fully recapitulate the dynamic extracellular matrix remodeling, vascularization, or endocrine signaling present in vivo. These factors play a crucial role in adipose tissue homeostasis and response to metabolic cues. Another limitation is the absence of a perfusable vascular network in the current model, which restricts nutrient and oxygen diffusion to the spheroid core. In vivo, adipose tissue is highly vascularized, and the lack of proper vascular integration in our spheroid cultures may affect long-term cell viability and metabolic function, particularly in larger spheroids. Additionally, while our study demonstrates enhanced spheroid retention and attachment using RGD-modified ELP coatings, further validation is needed to assess their long-term functional stability and the biological impact of RGD–integrin interactions on adipocyte differentiation and metabolic activity. Investigating the signaling pathways activated by RGD-mediated adhesion will be essential for understanding potential downstream effects on adipose tissue metabolism. Nevertheless, the standardized expression and purification methods used in this study ensure that the ELP sequences and their functional properties remain consistent across different experimental runs. Given the well-defined production and characterization protocols, this study provides a reproducible and adaptable approach for the further exploration of ELP-based biomaterials in various biomedical applications.

## 5. Conclusions

In this study, we successfully conjugated the C- and N-terminus of ELP with the cell adhesive peptide RGD without resulting in significant changes to the physical and chemical properties of the ELP construct. This study provided strong evidence that ELPs containing cell-binding RGD motifs can directly improve spheroid retention. To extend the application of these elastin-based coatings in adipose tissue culture, further biochemical experiments are needed to study integrin specificity as well as the respective signaling processes induced. The improved spheroid attachment and retention allowed by the ELP-(RGD)_3_-PEI will provide the possibility of extending the culture period for the physiologically relevant in vitro 3D spheroid model. The enhanced stability of adipocyte spheroids using ELP-(RGD)_3_-PEI coatings presents a promising avenue for advancing in vitro models for drug testing and obesity research. With a more physiologically relevant culture system, this platform could improve the prediction of anti-obesity drug screening and enable long-term studies on adipocyte metabolism and function under various therapeutic conditions. Compared to conventional 2D culture models, 3D spheroid models more accurately replicate in vivo-like adipocyte behavior, making them more suitable for studying adipose tissue remodeling, lipid metabolism, and the effects of pharmacological interventions. The ability to maintain spheroid integrity over extended culture periods is crucial for assessing the long-term efficacy and safety of potential therapeutics. Additionally, this improved 3D adipocyte culture model could be instrumental in studying metabolic diseases beyond obesity, such as diabetes and cardiovascular diseases, where adipose tissue dysfunction plays a critical role. These studies will provide a foundation for understanding the behavior of 3D adipocytes and help develop treatments that target the mechanistic causes of obesity and comorbidities.

## Figures and Tables

**Table 1:** Peptide sequences of the ELP and ELP-RGD constructs providing a comprehensive listing of the amino acid sequences.

Sample	Peptide Sequence
ELP	H_2_N–MVSACRGPG–[VGVPG]_40_–WP–COOH
(RGD)_3_-ELP	H_2_N–M(RGD)_3_VSACRGPG–[VGVPG]_40_–WPGGGGG–COOH
ELP-(RGD)_3_	H_2_N–MVSACRGPG–[VGVPG]_40_–WPGG(RGD)_3_GGG–COOH

**Table 2:** The OPA analysis confirmed that the ELP-(RGD)_3_ and (RGD)_3_-ELP were successfully conjugated to PEI.

Sample	%Conjugation
ELP-(RGD)_3_-PEI	11.1 ± 1.0
(RGD)_3_-ELP-PEI	12.0 ± 0.4

## Data Availability

The data presented in this study are available on request from the corresponding author.
